# Adenosquamous Cell Carcinoma Associated With Giant Lung Cyst: A Case Report

**DOI:** 10.7759/cureus.78340

**Published:** 2025-02-01

**Authors:** Ryusei Yoshino, Nanami Ujiie, Shunsuke Yasuda, Masahiro Kitada

**Affiliations:** 1 Thoracic Surgery and Breast Surgery, Asahikawa Medical University Hospital, Asahikawa, JPN

**Keywords:** adenosquamous cell carcinoma, emphysematous, lung cancer, lung cyst, surgery

## Abstract

Emphysematous lung cysts are considered to be a risk factor for the development of lung cancer, and there have been several reports of lung cancer cases that have developed in conjunction with large lung cysts. Emphysematous lung cysts are more common in heavy smokers, and it has been pointed out that early detection is difficult because the lung mass shadow overlaps with the lung cyst, so they are often detected in an advanced state. We report a case of this disease that was discovered incidentally. The patient was a 51-year-old man. During a health checkup, he was found to have a right lung cyst and a nodular shadow in his lung. He had a history of smoking 20 cigarettes a day for 30 years, and his Brinkman Index was 600. There were no other significant findings in the medical history. In addition to emphysema, a chest computed tomography (CT) scan revealed a large lung cyst and a lobulated, irregular nodular shadow measuring 15 x 14 mm in the S2 region of the right lung. A definitive diagnosis could not be made by transbronchial biopsy. Surgery was performed with the aid of a thoracoscope. A right upper lobectomy and mediastinal lymph node dissection were performed after a needle biopsy of the tumor was diagnosed as lung adenocarcinoma by intraoperative rapid diagnosis. The histopathological examination findings were diagnosed as adenosquamous cell carcinoma, WHO Grade 3, pT2apN0cM0. The patient is currently undergoing oral chemotherapy. In this case, it is important to detect large lung cysts using early chest CT scans. In addition, when performing surgery for lung cancer with a large lung cyst, it is necessary to be aware of the possibility of complications such as pneumothorax and peptic ulcer disease on the opposite side.

## Introduction

Emphysematous lung cysts are considered to be a risk factor for the development of lung cancer, and there have been reports of lung cancer cases that have developed in association with giant lung cysts [1]. Emphysematous lung cysts are more common in heavy smokers, and it has been pointed out that early detection is difficult because the lung mass shadow overlaps with the lung cyst, and that they are often detected in an advanced state [2]. In this report, we describe a case of this disease that was discovered incidentally.

## Case presentation

The patient was a 51-year-old man. He was 164 cm tall, weighed 69 kg, and had a BMI of 25.7 kg/m2. He had a history of smoking 20 cigarettes a day for 30 years, and his Brinkman Index was 600. He had no further past medical history. At a health checkup, he was found to have right lung cysts and lung nodules, and although a definitive diagnosis could not be determined at this time, the possibility of lung cancer was suspected, and he was referred to our department. On physical examination, his breath sounds were clear. There was also no enlargement of the lymph nodes in the neck or above and below the clavicles. Blood tests were all within normal limits, including tumor markers for lung cancer (Table 1). The respiratory function test showed a vital capacity of 4040 ml (109.2%) and a second volume of 2480 ml (62.9%), indicating mild obstructive disorder.

**Table 1:** Patient's laboratory results.

Blood collection items	Results	Normal range	Units
White blood cell	8.48	3.30-8.60	×10^3^/μl
Red blood cell	4.53	4.35-5.55	×10^6^/μl
Hemoglobin	13.9	13.7-16.8	g/dl
Platelet	338	158-348	×10^3^/μl
Blood urea nitrogen	11.6	8.0-20.0	mg/dl
Creatinine	0.83	0.65-1.07	mg/dl
Sodium	141	138-145	mmol/l
Potassium	4.3	3.6-4.8	mmol/l
Chloride	106	101-108	mmol/l
Asparate aminotransferase	15	13-30	U/l
Alanine aminotransferase	12	10-42	U/l
C-reactive protein	0.14	<0.15	mg/dl
Carcinoembryonic antigen (CEA)	4.7	<5.0	ng/ml
Cytokeratin 19 fragment (CYFRA)	2.05	<3.50	ng/ml
Pro-gastrin releasing peptide (ProGRP)	58.9	<81.0	pg/ml

**Figure 1:**
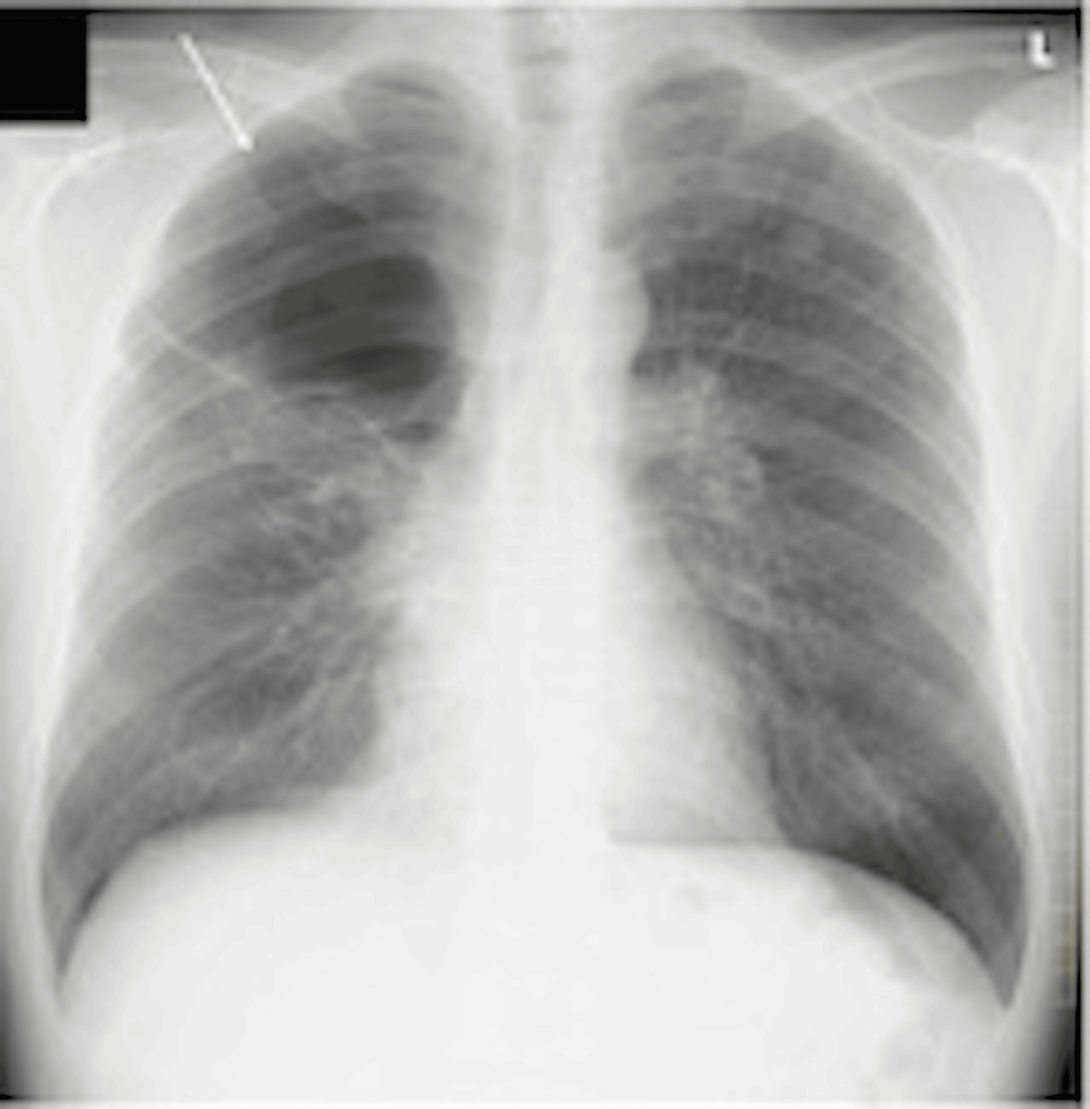
Chest radiograph (frontal view). Cystic lesion in the apex of the right lung.

Chest X-ray examination showed a cystic lesion in the right lung's apex (Figure 1). Chest computed tomography (CT) showed emphysema and an irregularly shaped nodule in the upper lobe of the right lung. There was no enlargement of the mediastinum, hilar, supraclavicular, or axillary lymph nodes. Fluorodeoxyglucose-positron emission tomography (FDG-PET) showed abnormal accumulation in the nodule in the right upper lobe with a maximum standardized uptake value of 12.2. Chest X-ray examination did not reveal any further abnormal findings.

**Figure 2:**
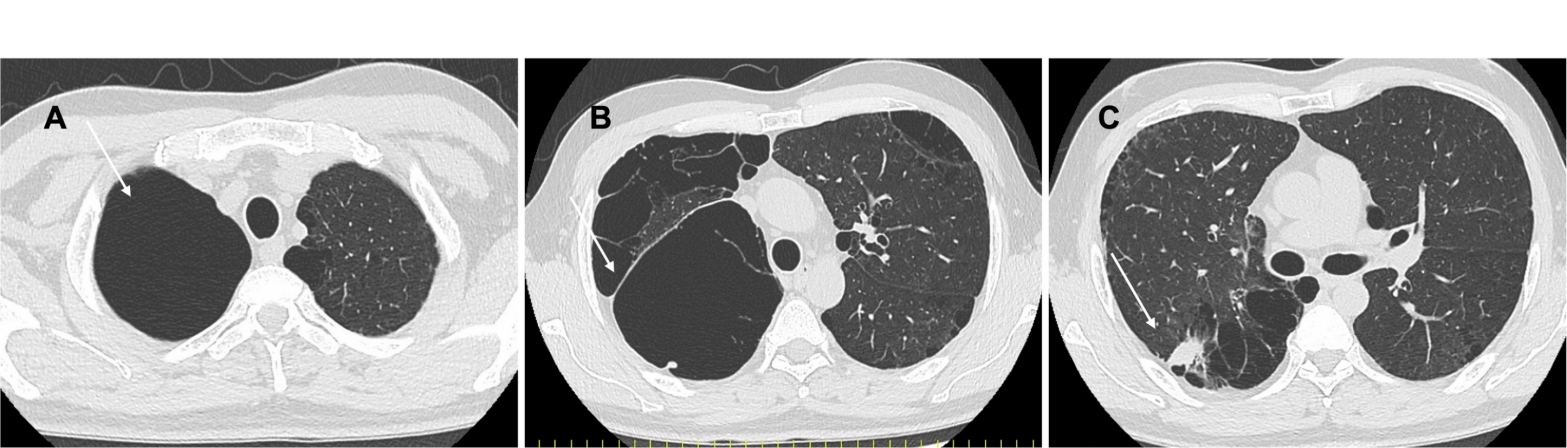
Chest computed tomography (CT) findings. A, B: Pulmonary cysts are present; C: A 15 x 14 mm irregular nodule was observed in the S2 upper lobe of the right lung.

Chest CT examination revealed an irregularly shaped nodule measuring 15 x 14 mm in the upper right lobe of the lung (Figure 2). A definitive diagnosis could not be made despite the performance of a bronchoscopic biopsy, and as lung cancer was suspected based on the imaging findings, surgical resection was recommended. 

**Figure 3:**
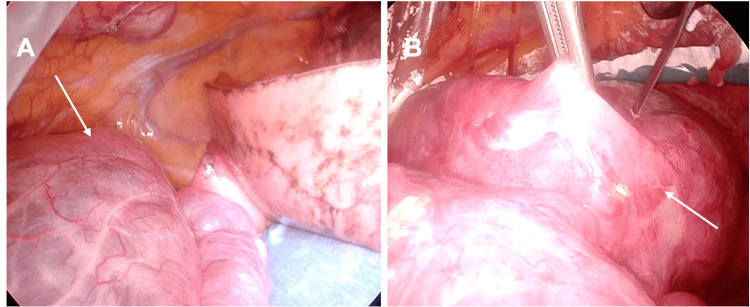
Intraoperative findings. A, B: Although the patient was under isolated lung ventilation, a giant lung cyst was observed. Surgery was initiated by incising and suturing the giant cyst wall to improve the visual field.

The surgery was performed with the aid of a thoracoscope. First, a needle biopsy was performed on the tumor, and it was diagnosed as adenocarcinoma by intraoperative rapid diagnosis. Therefore, the plan was to perform a right upper lobe resection and mediastinal lymph node dissection. The surgery involved opening and suturing the wall of the huge cyst to improve the field of vision (Figure 3). The remaining operation followed the standard procedure for right upper lobe resection. The operation took 125 minutes, and the amount of bleeding was 10 ml.

The pathological findings revealed adenosquamous cell carcinoma, WHO Grade 3, pT2apN0cM0, pStage1B. The background lung showed emphysematous changes, and in addition to the bulla that was visible to the naked eye, there were also scattered emphysematous cysts. The postoperative course was favorable. The chest tube was removed on the second postoperative day, and the patient was discharged on the seventh day. Postoperatively, the patient was treated with tegafur-gimeracil-oteracil potassium as an adjuvant therapy.

## Discussion

As patients with giant lung cysts are also at high risk of missing lung cancer or pneumothorax, it is important to detect them using chest CT scans. Lung cancer associated with lung cysts was first described by Bas and Singer in 1951, with varying growth patterns observed in these cases [3]. There are reports that classify them into the following types: type I nodules, which are in contact with the outer surface of the lung cyst; type II nodules, which arise from the cyst wall and protrude into the cyst cavity; type III cysts, which are wall thickening type, and type IV multiple cystic lesions, that include areas of soft tissue attenuation [4]. The type in which the tumor grows in the lung tissue adjacent to the cyst is the most common, as in this case. The mechanism of occurrence has been reported to involve squamous metaplasia of the cyst wall, carcinogenesis from cyst wall scarring, and the stagnation and accumulation of carcinogens within the cyst [5]. In this case, it is thought that chronic stimulation of the cyst wall may have been involved in carcinogenesis. In addition, it is thought that patients with lung cysts or emphysema are more likely to develop a pneumothorax because the structure of the lung becomes fragile [6]. In particular, in the case of giant lung cysts, rupture of the cyst is often the direct cause. Therefore, in patients with giant lung cysts, the risk of overlooking lung cancer or pneumothorax is also high, so it is important to detect them early by using a chest CT. 

In addition, based on this case, it is necessary to be careful during the surgical period for lung cancer with a large lung cyst, keeping in mind the possibility of complications such as pneumothorax on the opposite side and peptic ulcers [7]. In general, surgical indications for giant lung cysts include those that exhibit progressive enlargement, those where re-expansion of the compressed lung is anticipated, and those associated with respiratory symptoms [7]. Previous reports have documented complications such as contralateral pneumothorax during surgery for giant lung cysts, as well as challenges in anesthesia management for the combined resection of giant lung cysts and lung cancer following COVID-19 pneumonia [8,9]. Therefore, it is important to keep in mind the possibility of contralateral lesions when the respiratory condition worsens during or after surgery. Furthermore, chronic obstructive pulmonary disease is attracting attention as a chronic systemic inflammatory syndrome, which may increase the frequency of complications such as peptic ulcer, so attention and care may be needed for postoperative complications [10,11]. Therefore, when surgery is considered for lung cancer with a giant lung cyst, it is necessary to be aware of the possibility of complications such as pneumothorax on the opposite lung and peptic ulcer disease.

In terms of the actual surgery, in this case, we started by improving the field of vision by debulking the cyst wall under thoracoscopic assistance, which was effective. A study on surgical resection in cases of giant pulmonary emphysema reported that while video-assisted thoracic surgery is a safe and effective treatment option, postoperative air leaks may still occur [12]. On the other hand, a study reported that the operative risk could escalate due to the patient's history of smoking, so the choice of a surgical procedure needs to be carefully considered for each individual patient [12]. In this case, a port was initially created to observe the chest cavity; however, due to a poor field of view, a small thoracotomy was performed to minimize the risk of complications.

## Conclusions

This case highlights the importance of early detection of giant emphysematous lung cysts and associated lung cancer by obtaining chest CT scans, particularly in patients with a history of heavy smoking. Early diagnosis is crucial, as overlapping lung mass shadows with cysts can complicate detection. Surgical management of lung cancer in the presence of large cysts requires meticulous planning to prevent complications, such as contralateral pneumothorax and peptic ulcers.

In this case, the surgical approach, including the initial incision and suturing of the cyst wall under thoracoscopic assistance, proved effective in securing visibility and minimizing complications. This technique may be particularly beneficial in similar cases involving giant lung cysts. Moreover, the findings emphasize the need for personalized surgical strategies depending on each patient's medical history and risks. Finally, this report underscores the necessity for ongoing postoperative care, including monitoring for systemic inflammatory complications and appropriate adjuvant therapies, to improve patient outcomes. Future studies on the optimal management of lung cancer associated with giant cysts are warranted to establish standardized protocols for these complex cases.
